# Assessing the Dream-Lag Effect for REM and NREM Stage 2 Dreams

**DOI:** 10.1371/journal.pone.0026708

**Published:** 2011-10-26

**Authors:** Mark Blagrove, Nathalie C. Fouquet, Josephine A. Henley-Einion, Edward F. Pace-Schott, Anna C. Davies, Jennifer L. Neuschaffer, Oliver H. Turnbull

**Affiliations:** 1 Department of Psychology, Swansea University, Swansea, Wales, United Kingdom; 2 Department of Psychiatry, Massachusetts General Hospital, Harvard Medical School, Boston, Massachusetts, United States of America; 3 Department of Psychology, University of Massachusetts, Amherst, Massachusetts, United States of America; 4 School of Psychology, Bangor University, Bangor, Wales, United Kingdom; Royal Holloway, University of London, United Kingdom

## Abstract

This study investigates evidence, from dream reports, for memory consolidation during sleep. It is well-known that events and memories from waking life can be incorporated into dreams. These incorporations can be a literal replication of what occurred in waking life, or, more often, they can be partial or indirect. Two types of temporal relationship have been found to characterize the time of occurrence of a daytime event and the reappearance or incorporation of its features in a dream. These temporal relationships are referred to as the day-residue or immediate incorporation effect, where there is the reappearance of features from events occurring on the immediately preceding day, and the dream-lag effect, where there is the reappearance of features from events occurring 5–7 days prior to the dream. Previous work on the dream-lag effect has used spontaneous home recalled dream reports, which can be from Rapid Eye Movement Sleep (REM) and from non-Rapid Eye Movement Sleep (NREM). This study addresses whether the dream-lag effect occurs only for REM sleep dreams, or for both REM and NREM stage 2 (N2) dreams. 20 participants kept a daily diary for over a week before sleeping in the sleep laboratory for 2 nights. REM and N2 dreams collected in the laboratory were transcribed and each participant rated the level of correspondence between every dream report and every diary record. The dream-lag effect was found for REM but not N2 dreams. Further analysis indicated that this result was not due to N2 dream reports being shorter, in terms of number of words, than the REM dream reports. These results provide evidence for a 7-day sleep-dependent non-linear memory consolidation process that is specific to REM sleep, and accord with proposals for the importance of REM sleep to emotional memory consolidation.

## Introduction

It is well-known that events and memories from waking life can be incorporated into dreams [1]. These incorporations can be a literal replication of what occurred in waking life, or, more often, they can be partial or indirect. For example, in dreams that are spontaneously recalled at home, there is a literal replay of waking life events in just 1–2% of the dream reports, but 65% of the reports reflect aspects of recent waking life experiences [2]. Two types of temporal relationship have been found to characterize the time of occurrence of a daytime event and the reappearance or incorporation of its features in a dream [3]–[7]. These relationships are referred to as (1) the day-residue or immediate incorporation effect, where there is the reappearance of features from events occurring on the immediately preceding day [3]–[9], and (2) the dream-lag effect, where there is a reappearance of features from events occurring 5–7 days prior to the dream [3]–[7]. Nielsen et al. [3] note that the two effects are curvilinear in nature such that, when plotted together over a time line of 1 week, they form a U-shaped curve.

The dream-lag effect was investigated by Nielsen et al. [3] using a between-subjects design in which each participant was randomly assigned to one of 7 groups; these had a period of from 1 to 7 days between a comparison day and the occurrence of a dream that the participant reported. Participants rated the level of correspondence between the dream report and their report of the events of the comparison day. The authors found a significantly higher level of rated correspondence between waking life experiences and dream reports when those experiences occurred 1–2, or 5–7 days before the dream, in comparison to when the experiences occurred 3–4 days before the dream. Nielsen et al. [3] thus confirmed the dream-lag effect and suggested that it is evidence for an approximately 7 day period of memory consolidation. Blagrove et al. [10] used a within-subjects design in which participants kept a daily diary and a dream diary for 14 days. This design resulted in each participant having many instances of a dream report that could be compared to the events of the day before, and many instances of dream reports that could be compared to events of 2, or 3, etc., days before the dream. Participants rated the level of correspondence between every one of their dream reports and every daily diary record. Significant day-residue and dream-lag effects were found, as well as a decrease in level of correspondence between dream reports and diary records when dreams occurred 8 or more days after the comparison day.

It has been claimed that a possible confounding factor here is that there may be an influence of recurrent routine events that lead to apparently delayed incorporations, because a person dreaming about an event from the day before, but, with the event recurring each week, may give a spurious correspondence of that dream report with the diary record of the same day of the week, but a week earlier. However, Nielsen et al. [3] did take account of this possibility by removing periodic events from consideration in their process of comparing diary records and dream reports. Furthermore, Blagrove et al. [10] tested for this potential confound by the novel technique of assessing the level of correspondence between dream reports and diary records from the same day of the week as the day before the dream. Scores for comparing dream reports with diary records for the day exactly a week before, or exactly a week after the day before the dream were low: there was thus no evidence for a weekly periodic confound.

The physiological basis for the dream-lag effect is suggested as being due to the relocation of memories from the hippocampus to the neocortex over a time period of approximately one week after initial learning [11]. Regarding this relocation, Walker [12] proposes that sleep firstly strengthens individual memory items, and then, over a longer time course, connects memories together. He states that this produces general and abstract knowledge, and even creative combinations of individual memories, by a process of reactivation of memories during sleep. It has been suggested that dream content may be reflective of the neural activity behind memory consolidation during sleep [11], [13]–[19]. Experimental evidence for the link between memory consolidation and dream imagery has been reviewed by Wamsley and Stickgold [19]. The evidence includes the finding that improved performance at retest on a virtual maze navigation task was strongly associated with dream imagery about that task [20]. From this evidence Wamsley and Stickgold propose that “even within a single dream experience, sleep mentation reflects the interleaved reactivation of memory fragments from different recent and remote sources, allowing newly acquired information to become increasingly connected with related memory traces across time.” [19]

The dream-lag studies cited above [3]–[7], [10] used spontaneous home recalled dream reports, which can be from Rapid Eye Movement Sleep (REM) or non-Rapid Eye Movement Sleep (NREM), although more frequently from REM [21]. The question thus arises of whether the dream-lag effect occurs only for REM dreams, or for both REM and NREM dreams. The basis for suggesting that REM and NREM dreams may differ in this regard is the importance of REM sleep for emotional memory consolidation [22]–[24]. A theoretical account for a specifically REM sleep memory consolidation function is provided by Walker and Stickgold [25], who write: “We propose that a first post-encoding stage, which might occur preferentially during SWS [slow wave sleep, or N3, a part of NREM], consolidates new episodic item memories while keeping individual memory representations separate and distinct. By contrast, a second, potentially REM-dependent, stage supports the integration of these and older memories into rich associative networks… It is this second stage of memory integration that extracts, abstracts and generalizes recently consolidated item memories in a process that might be linked to the production of dreams.” They also state that the integration of new with old memories may occur across multiple nights. We propose that if the dream-lag effect is a result of this integrative second stage, or of REM-dependent emotional memory processing, then the dream-lag effect might be found only for REM dreams. Following Walker and Stickgold [25], we also predict that delayed incorporation dreams are more likely to occur later in the night than earlier.

To summarize, this present study addresses whether the dream-lag effect occurs only for REM dreams, or for both REM and NREM dreams. For this study NREM stage 2 (N2) is assessed rather than NREM stage 3 (N3) so that NREM dreams can be collected at similar times of night as the REM dreams, given that REM predominates later in the night, that N3 predominates during the early part of the night, and that N2 occurs across the night.

## Results

There were 76 REM and 66 N2 awakenings. From these awakenings, 59 REM and 22 N2 dream reports with word count of at least 20 words were obtained. After the initial 80 min of uninterrupted sleep, the first dream report of the night occurred from REM on 21 occasions, and from N2 on 13 occasions. Toward the end of the night's sleep, after 8 hours since sleep onset (SSO), there were far more REM than N2 dreams, with 11 REM dream reports and only 1 N2 dream report. The mean time since sleep onset for the two categories of dreams were: REM dreams, mean = 6.16 hrs SSO (SD = 1.77); N2 dreams, mean = 4.33 hrs SSO (SD = 2.33).

19 participants provided at least one REM dream report (mean number of REM dream reports per participant = 3.05 (SD = 1.96)). 13 participants provided at least one N2 dream report (mean number of N2 dream reports = 1.69 (SD = .75)). Although there was a potential maximum time period of 11–12 days between diary records and dream reports, some participants had a lower maximum time period due to not having recalled a dream on the second laboratory night. All participants did provide data for ratings of correspondence between dream reports and diary records from 1 to 9 days earlier, and it is hence only these 1–9 days' data that are analysed here. For inferential statistics, the data from the separate days are combined for each participant into periods between diary record and dream of 1–2 days, 3–4 days, 5–7 days (these 3 combined periods follow the analysis of Nielsen et al. [3]) and 8–9 days. That a dream-lag period of specifically 5–7 days is hypothesised follows from Nielsen et al. [3] and Blagrove et al. [10], and this 5–7 days definition is strictly adhered to so as to avoid multiple comparisons and thus to minimise the possibility of type 1 errors.

**Figure 1:**
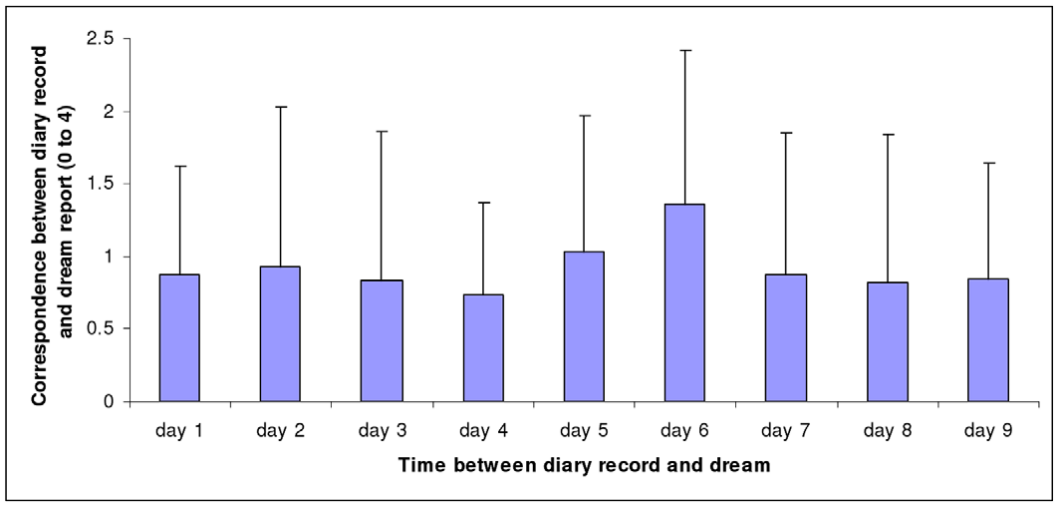
Correspondence between REM dream reports and diary records as a function of time. Mean correspondence scores (and Standard Deviations) between REM dream reports and diary records as a function of time between diary day and dream.

**Figure 2:**
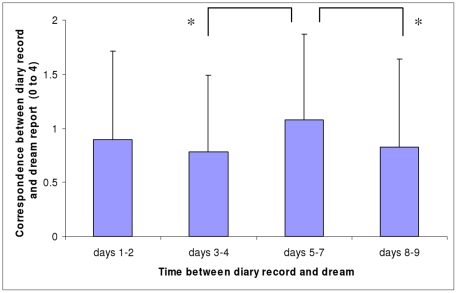
Correspondence between REM dream reports and diary records as a function of time period. Mean correspondence scores (and Standard Deviations) between REM dream reports and diary records as a function of time period between diary day and dream. * p≤.05 (Wilcoxon test).

Figure 1 shows the mean correspondence scores between REM dream reports and the diary records of each of the previous 9 diary days. Inferential statistics are not conducted on these data, but are instead conducted on the mean period data, as shown in Figure 2.

Figure 2 shows that the mean correspondence scores for REM dreams differed significantly across the 4 time periods (Friedman test, chi sq (df = 3) = 8.22, p<.05). As hypothesised, the mean correspondence score for days 5–7 was significantly higher than for days 3–4 (Wilcoxon test, z = 2.07, p = .039). The days 5–7 mean correspondence score was also higher than for days 8–9 (Wilcoxon test, z = 1.94, p = .052).

**Figure 3:**
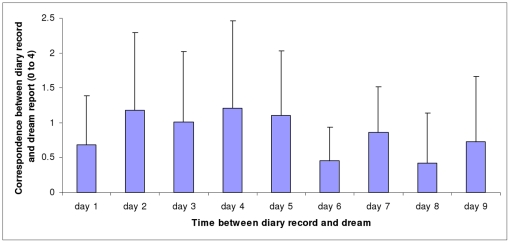
Correspondence between N2 dream reports and diary records as a function of time. Mean correspondence scores (and Standard Deviations) between N2 dream reports and diary records as a function of time between diary day and dream.

**Figure 4:**
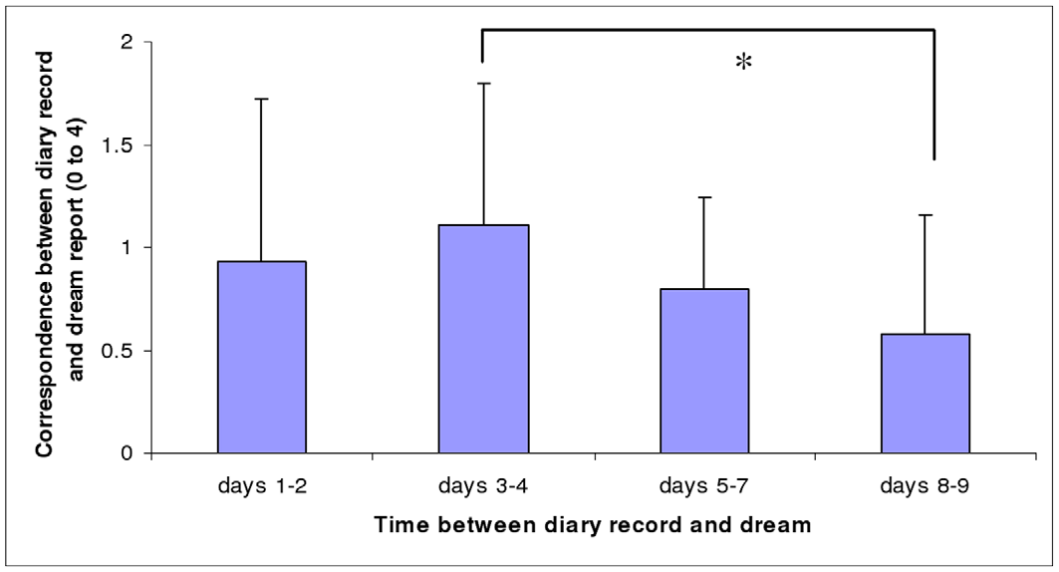
Correspondence between N2 dream reports and diary records as a function of time period. Mean correspondence scores (and Standard Deviations) between N2 dream reports and diary records as a function of time period between diary day and dream. * p<.05 (Wilcoxon test).

Figure 3 shows the mean correspondence scores between N2 dream reports and the diary reports of each of the previous 9 diary days. Inferential statistics are not conducted on these data, but are instead conducted on the mean period data, as shown in Figure 4.

Figure 4 shows that the mean correspondence scores for N2 dreams did not differ significantly across the 4 time periods (Friedman test, chi sq (df = 3) = 5.92, n.s.). However, the mean correspondence score for days 3–4 was significantly higher than for days 8–9 (Wilcoxon test, z = 2.15, p = .032).

The 5–7 day dream-lag effect was thus found for REM but not N2 dream reports. However, these results are confounded by dream report length, in that the mean total recall count (length in words) of the 59 REM dream reports = 147.39 words (SD = 137.18), exceeds the mean total recall count of the 22 N2 dream reports = 86.14 words (SD = 89.51). Therefore, rather than the dream-lag effect being specific to REM dream reports, it may instead be a function of dream report length.

To establish whether the dream-lag is only found for longer dream reports a further analysis was undertaken. For the 59 REM dream reports, a median split around the median total recall count was performed. Three dream reports were on the median of 120 words: two sub-samples were thus produced, REM dream reports with length in words below the median (n = 29), and REM dream reports with length in words above the median (n = 27). The dream-lag calculations described above were then computed for these two sub-samples separately; the number of participants in each analysis was 16 for the low total recall count dream reports and 10 for the high total recall count dream reports. For both sub-samples the mean correspondence scores followed the same dream-lag pattern as for the full sample (i.e., correspondence scores for days 5–7 > days 8–9 > days 3–4). Means: shorter dream reports, days 1–2 = 0.82; days 3–4 = 0.62; days 5–7 = 1.15; days 8–9 = 0.71; longer dream reports, days 1–2 = 0.69; days 3–4 = 0.64; days 5–7 = 0.96; days 8–9 = 0.76. Importantly, the pattern was *not* more significant for the longer dream reports in comparison to the shorter dream reports (shorter dream reports - Friedman test, chi sq = 7.71, df = 3, p = .053; Wilcoxon tests, days 5–7 > days 3–4, z = 2.27, p = .023; days 5–7 > days 8–9, z = 1.76, p = .078. Longer dream reports - Friedman test, chi sq = 1.71, df = 3, p>.1; paired comparisons not significant). Thus, total length of dream report in words does not appear to confound the finding of the dream-lag being present for REM but not N2 dream reports.

To establish whether the dream-lag effect is present more for dreams later in the night than for dreams earlier in the night a further analysis was undertaken. The median time since sleep onset for the 59 REM dream awakenings was found to be 6.39 hrs. A median split around this time was performed; this produced 29 REM dreams from before and 29 REM dreams from after the median time SSO. The mean times SSO for the two sub-samples were: earlier REM dreams, mean time SSO = 4.65 hrs (SD = 1.13); later REM dreams, mean time SSO = 7.67 hrs (SD = 0.72).

The number of participants for each sub-sample was 17 for the earlier REM dreams, and 15 for the later REM dreams. The dream-lag calculations described above were computed for these two sub-samples separately. The dream-lag pattern was found to be significant for the earlier REM dreams (Means: days 1–2 = 1.21; days 3–4 = 0.89; days 5–7 = 1.27; days 8–9 = 0.90: Friedman test, chi sq = 6.87, df = 3, p = .076; Wilcoxon tests, days 5–7 > days 3–4, z = 2.29, p = .022; days 5–7 > days 8–9, z = 2.06, p = .039), but was not significant for the later REM dreams (Means: days 1–2 = 0.52; days 3–4 = 0.59; days 5–7 = 0.83; days 8–9 = 0.66: Friedman test, chi sq = 1.86, df = 3, p>.1, paired comparisons not significant).

To establish whether there might be some overall difference between REM and N2 dream reports for their correspondence scores, the mean correspondence scores for days 1 to 9 were calculated for REM and N2 dream reports separately. These were, for REM dream reports, mean = 0.92 (SD = 0.68, n = 19), and for N2 dream reports, mean = 0.85 (SD = 0.44, n = 13). For the 12 participants who had at least 1 REM dream report and at least 1 N2 dream report, the difference between correspondence scores was non-significant (REM dream reports, mean = 0.76 (SD = 0.55), N2 dream reports, mean = 0.85 (SD = 0.46); Wilcoxon test, z = 0.71, n.s.). A similar comparison was made between the correspondence scores for the earlier and the later REM dreams. The mean correspondence scores were computed for all participants who had at least one earlier (pre-median time SSO) REM dream and at least one later (post-median time SSO) REM dream (number of participants = 13). These means did not differ significantly (mean correspondence across days 1 to 9 for earlier REM dreams = 0.96 (SD = 0.83), mean correspondence for later REM dreams = 0.68 (SD = 0.51); Wilcoxon test, z = 1.43, p = .15).

The day 1 correspondence scores were clearly low. As described in the Method, these scores underestimate the true level of day-residue incorporations as they do not allow for the scoring of correspondences between dream reports that have reference to the laboratory experience and the waking life experience of being in the laboratory, because accounts of the latter were not included in the diary records. The REM and N2 dream reports were assessed for the presence of direct or indirect references to the experimental procedure by 2 judges (MB and J H-E) using the Experimental Relatedness Scale [26]. Ten of the REM dream reports and 7 N2 dream reports were judged to refer to the laboratory or experimental conditions, such as by having content related to the rooms in which the study occurred, the equipment, the study requirements or the researchers. The day 1 correspondence scores would thus be higher if these correspondences were included.

The dream-lag analyses were repeated after exclusion of these laboratory incorporation dream reports. This reduced the number of dream reports to 49 for REM and 15 for N2. For the REM analyses, number of participants remained at 19; for the N2 analyses, number of participants = 12. The removal of these dream reports made negligible difference to the mean correspondence scores for day 1 or for days 1–2 combined. Only small differences were made to the other means; when inferential statistics were applied to these data the correspondence score for days 5–7 was found to be significantly greater than for days 3–4 and days 8–9 for the REM dream reports (both ps<.05, zs = 2.33 and 2.02 respectively), and for the N2 dream reports the difference in correspondence scores between days 3–4 and days 8–9 was found to be no longer significant (z = 1.69).

## Discussion

Previous work on the delayed incorporation of waking life events and memories into dreams has used dream reports collected after spontaneous awakenings at home. The sleep stage at awakening has thus been unknown. However, as most such dreams are likely to have been from REM sleep, it was a realistic assumption that the dream-lag effect occurs at minimum for REM dreams. At issue was whether it also occurs for NREM dreams. The present study shows that the dream-lag effect occurs for REM dreams but not N2 dreams. These results provide evidence for a 7-day sleep-dependent memory consolidation process that is *specific* to REM sleep, and accord with proposals for the importance of REM sleep to emotional memory consolidation. The dream-lag effect also suggests a complex non-linear memory reactivation function, which is supported by Medina et al.'s review [27], which concludes that there may be several phases of memory consolidation due to recurrent rounds of protein synthesis necessary to permanently store new information. Not known, however, is whether a N2 dream-lag effect and non-linear reactivation could be found for learning tasks whose consolidation has been shown to be dependent on NREM sleep, such as maze learning [20] or learning on paired-associates and simple motor tasks such as the pursuit rotor [28].

We acknowledge the potential confound in the study that the N2 dream reports were shorter than the REM dream reports, as would be expected from the previous literature [29]. However, there are three reasons to doubt that this confound accounts for the difference between REM and N2 dreams on the dream-lag effect. Firstly, with a mean length of 86 words the N2 dreams were not especially brief. Secondly, there was no significant overall difference between the REM and N2 dream reports on their mean correspondence scores with diary records. And, thirdly, REM dream reports of below median length in words actually showed a greater dream-lag effect than did the REM dream reports of above median length. The latter finding may be because the longer dreams are more elaborated, and hence may have more details that are distant from, or that cannot be matched to any waking life event. This possibility follows from Foulkes & Schmidt's finding that “longer reports are not so much collections of *more* dream fragments on the order of the shorter reports as they are *extensions* of such fragments into longer narrative units.” [30] [Italics in original.]

The prediction that delayed incorporation of waking life events would occur more for dreams later in the night was not confirmed. Although there was a greater correspondence for days 5–7 than for days 3–4 and days 8–9 for the earlier and the later REM dream sub-samples, the differences between days 5–7 and days 3–4, and between days 5–7 and days 8–9, were only significant for the earlier REM dreams. Indeed, the mean correspondence of dream reports with diary records across all the periods (i.e., days 1–9) was greater for the earlier than for the later REM dreams. This may be because a progressive decrease in direct references to waking life and increased abstraction occurs with increased duration of sleep [31]–[33]. We thus recommend that future investigations of the dream-lag effect assess the dreams of early and middle REM periods, rather than just the generally later, home spontaneous awakening dreams used in the previous literature.

We acknowledge a second potential confound in the study, in that the mean time SSO for the REM dreams was greater than for the N2 dreams. However, as the earlier REM dreams showed a larger dream-lag effect than did the later REM dreams, and as the mean time SSO for the earlier REM dreams (4.65 hrs) was very similar to the mean time SSO for the N2 dreams (4.33 hrs), the length of time since sleep onset that the dreams occur does not appear to be an explanation for the finding that the dream-lag is present for REM but not N2 dreams.

There is now a considerable literature indicating that some aspects of memory consolidation occur during sleep [27], [28], [34]–[40]. However, little experimentation on this has been performed in humans across multiple nights. We therefore reiterate Nielsen et al.'s suggestion [3] that, in addition to the dream-lag effect being evidence, in humans, for a specific 5–7 day component for memory consolidation, future research should investigate the possibility that later (5th to 7th night) memory processing is qualitatively different from the immediate (1st and 2nd night) processing. This would entail comparing the characteristics of delayed incorporations of memory elements and waking life events into dreams with the characteristics of incorporations for time periods earlier and later than 5–7 days. In conducting such an analysis, Nielsen et al. [3] found that delayed incorporations had significantly greater prevalence of problem resolution, of positive emotions and interpersonal interactions than did immediate incorporations. They interpreted this as supporting a socio-emotional memory consolidation function for sleep that takes place 5–7 days after waking life events. An alternative possibility for a difference between immediate and delayed memory processing during sleep is Walker's proposal [12] that, over time, sleep reduces the felt emotional component in memories, leaving instead just the knowledge that an emotion was present. We suggest that dream content might be used to test Walker's proposal, in that delayed dream incorporations would be predicted to have lower emotional load than would immediate incorporations. This investigation of the characteristics of incorporations of waking life events into dreams would require that participants identify which parts of each dream report, and which parts of each daily diary record, they consider correspond with each other. This detailing of where correspondences are identified as occurring in the reports would be an improvement on the design of the current study, which did not require the particular correspondences to be recorded. This detailing of correspondences was not done in the current study because of the high workload commitment already asked of the participants, which resulted in a maximum number of 120 separate dream report and daily diary record comparisons for one participant.

In summary, we have shown that the dream-lag effect occurs for REM but not N2 dreams. These results point to a memory consolidation function or mechanism that is specific to REM sleep. The importance of such a mechanism is shown by suggestions of a connection between sleep quality and mood disorders, and that mood disorders and nightmares can result if emotional memory processing does not occur during sleep [12], [24]. In addition to pointing toward such a sleep-dependent memory consolidation function, the content of immediate and delayed incorporation dreams might be used to test theories of the characteristics of memory processing across time during sleep. Such work would respond to the conclusion by Rasch and Born that, whereas the reactivation of memories during sleep has been ‘compellingly demonstrated’, the nature of the information extracted in this process is currently unclear [41].

## Materials and Methods

20 participants (10 males, 10 females; mean age = 20.5 (SD = 2.0)) kept a daily diary for over a week before sleeping in the sleep laboratory for 2 nights, these 2 nights being separated by one non-laboratory night. All participants began their daily diaries on the same day, the Sunday of the week prior to the pre-arranged sleep laboratory week, and then slept for two nights in the sleep laboratory on the next week, either Monday and Wednesday nights or Tuesday and Thursday nights.

In the laboratory, sleep was monitored by polysomnography with electrodes at: F4 and C4 for EEG, applied according to the standard 10–20 system; above right outer canthus and below left outer canthus for EOG-detected eye movements; left and right mastoids for reference, and on the chin for electromyography (EMG). Sleep scoring followed the AASM Manual for the Scoring of Sleep [42]. Awakenings were not scheduled to occur during the first 80 minutes of sleep. The first awakening was scheduled from the first stage 2 period (N2) after the first 80 minutes of sleep, then from the next REM period, and thereafter whenever 10 minutes of either REM or N2 were obtained. (Confirmatory sleep scoring was conducted later by a second scorer.) Awakenings were conducted at the end of 10 minutes of either REM or N2 sleep. When the stage criteria were met, the participant was awoken by a buzzer system. After the participant turned off the buzzer they were given the verbal prompt ‘Was anything going through your mind before you were woken?’ If they could remember a dream they then recorded a report of it into a Digital Voice Recorder (Olympus VN-2100PC). Recordings were given a random identifying number and each morning were sent by email to a researcher blind to the awakening conditions and blind to details of the participants. This researcher transcribed each dream report.

One week after the second sleep laboratory night each participant was provided with a randomised set of their own diary records, and a randomised set of their transcribed dream reports, and were asked to rate the level of correspondence between every dream report and every diary record using the following scale: 0 = none; 1 = weak; 2 = moderate; 3 = high; 4 = extremely high.

The ratings were recorded using a matrix as described by Blagrove et al. [10]. An example portion of a matrix can be found in Table 1.

**Table 1:** Example portion of a matrix used to record correspondence ratings (0–4) between dream reports and diary records.

Diary Day	Dream 443	Dream 547	Dream 621	Dream 998
3				
10				
2				
6				
5				
9				
…..				

The instructions given to participants for this task were:

“Please enter a number from 0 to 4 into each of the cells in the column for that dream to show how much correspondence there is between the dream and each diary day. Once you have finished that column please move on to the next column; so, you then read the next dream and then look at all the diary entries in turn, again entering a number 0 to 4 in each cell. Again, the emphasis is on rating how much correspondence a given dream has with each and every diary entry.”

After the matrix was completed and returned to the experimenters, the level of matching between dream reports and diary records was computed as a function of number of days between diary day and dream report, for REM and N2 dreams separately. When the dream report is compared to the diary record of the day before the night of the dream, this time period is termed Day 1, the period for a comparison with the day before that day is termed Day 2, and so on. Only dream reports of at least 20 words were included in the analyses. Report length in words (termed total recall count) was calculated following Antrobus' definition [43]: “the count of all words in sentences or phrases in which the subject was describing something that had occurred just before waking. It excluded ‘ahs,’ ‘uhms,’ repeated and corrected words, and all commentary on the experience, the report, or the current status of the subject.”

A confounding problem for the correspondence rating procedure arises because of the occasional incorporation of the laboratory experience into dreams. According to Schredl's meta-analysis [44], 19% of dreams collected in the sleep laboratory have direct references to the laboratory or the experimental procedure, and 38% have a direct or an indirect reference. It is not feasible to determine whether such laboratory references occur solely due to the memory of the pre-sleep preparation procedures and laboratory environment, or due to being asleep in bed in the laboratory at the time the dream occurs, or to both of these factors. It was necessary to avoid confounding the diary record / dream report comparison procedure with such laboratory incorporations that might have been stimulated because of being asleep in the laboratory at the time that the dream occurs, rather than stimulated solely by the memory of the pre-sleep experiences. Therefore, on sleep laboratory nights participants completed their diaries before attending the sleep laboratory; the dream report would then be compared only to the experiences of the day prior to being in the laboratory. This procedure of not recording sleep laboratory events in the diary necessarily leads to the underestimation of the incorporation of recent (day 1) events into dreams, as it disallows any matches of laboratory related dream content to a diary record of the experiences of being in the sleep laboratory. However, this day 1 correspondence level is not needed for the identification of the dream-lag, the latter just requiring the days 5–7 correspondence score to be greater than that for days 3–4 and greater than the correspondence score for days 8 and higher.

### Ethics statement

The study was approved by the Research Ethics Committee of the Department of Psychology, Swansea University. All participants gave written informed consent and were treated in accordance with the principles expressed in the Declaration of Helsinki.
